# Effect of fruit restriction on glycemic control in patients with type 2 diabetes – a randomized trial

**DOI:** 10.1186/1475-2891-12-29

**Published:** 2013-03-05

**Authors:** Allan S Christensen, Lone Viggers, Kjeld Hasselström, Søren Gregersen

**Affiliations:** 1 Department of Nutrition, Regional Hospital West Jutland, Jutland, Denmark; 2 Medical Department, Regional Hospital West Jutland, Jutland, Denmark; 3 Department of Endocrinology and Metabolism, Aarhus University Hospital, Aarhus, Denmark

**Keywords:** Glycemic control, Nutrition counseling, Weight loss, Fruit, Medical nutrition therapy, Type 2 diabetes

## Abstract

**Background:**

Medical nutrition therapy is recognized as an important treatment option in type 2 diabetes. Most guidelines recommend eating a diet with a high intake of fiber-rich food including fruit. This is based on the many positive effects of fruit on human health. However some health professionals have concerns that fruit intake has a negative impact on glycemic control and therefore recommend restricting the fruit intake. We found no studies addressing this important clinical question. The objective was to investigate whether an advice to reduce the intake of fruit to patients with type 2 diabetes affects HbA_1c,_ bodyweight, waist circumference and fruit intake.

**Methods:**

This was an open randomized controlled trial with two parallel groups. The primary outcome was a change in HbA_1c_ during 12 weeks of intervention. Participants were randomized to one of two interventions; medical nutrition therapy + advice to consume at least two pieces of fruit a day (high-fruit) or medical nutrition therapy + advice to consume no more than two pieces of fruit a day (low-fruit). All participants had two consultations with a registered dietitian. Fruit intake was self-reported using 3-day fruit records and dietary recalls. All assessments were made by the “intention to treat” principle.

**Results:**

The study population consisted of 63 men and women with newly diagnosed type 2 diabetes. All patients completed the trial. The high-fruit group increased fruit intake with 125 grams (CI 95%; 78 to 172) and the low-fruit group reduced intake with 51 grams (CI 95%; -18 to −83). HbA_1c_ decreased in both groups with no difference between the groups (diff.: 0.19%, CI 95%; -0.23 to 0.62). Both groups reduced body weight and waist circumference, however there was no difference between the groups.

**Conclusions:**

A recommendation to reduce fruit intake as part of standard medical nutrition therapy in overweight patients with newly diagnosed type 2 diabetes resulted in eating less fruit. It had however no effect on HbA_1c_, weight loss or waist circumference. We recommend that the intake of fruit should not be restricted in patients with type 2 diabetes.

**Trial registration:**

http://www.clinicaltrials.gov; Identifier: NCT01010594.

## Background

The prevalence of type 2 diabetes (T2DM) is still rising and has reached epidemic proportions in most countries [1]. It is estimated that around 350 million people worldwide have T2DM [1]. Individuals with T2DM have increased morbidity and mortality and represent a huge economic burden for society. The importance of medical nutrition therapy (MNT) is recognized as one of the cornerstones in the treatment of T2DM [2-4]. Several evidence-based nutrition guidelines have been published and show that both diet quality and quantity have a huge impact on T2DM [3-5]. A variety of fibre-rich food like fruit and vegetables are generally recommended [4,5].

Fruit contain a wide range of specific bioactive substances which can act through multiple pathways in the human body e.g. as antioxidants, reduce inflammation and improve endothelial function [6-8]. High fruit intake has been shown to reduce the risk of e.g. cardiovascular disease [9,10] and some cancer types [11].

Health professionals often have concerns about the sugar content of fruit and therefore advice individuals with T2DM to restrict their intake to a maximum of two pieces a day. Few studies have addressed whether high fruit intake is associated with glycemic control and these have shown either no association [12-15] or an inverse association [16] between fruit intake and either HbA_1c_ or blood glucose. However, these are all observational studies and none are performed in subjects with T2DM. Therefore sparse data exist to answer the question whether or not fruit has a negative impact on blood glucose levels in subjects with T2DM. We carried out this study to test the hypothesis that an advice to restrict fruit intake to a maximum of two pieces a day compared to at least two pieces a day results in an improved glycemic control in adults with type 2 diabetes.

## Methods

### Subjects

Volunteers were selected from patients referred to MNT by their GP to the Outpatient Clinic at Department of Nutrition, Regional Hospital West Jutland. Eligible patients were adults with T2DM (duration of T2DM < 12 months), HbA_1c_ values 12.0% or less and who accepted to adhere to the protocol. Exclusion criteria were clinically significant cardiovascular, renal or endocrine disease. A total of 136 subjects were invited and 63 subjects were randomized. Each subject gave informed, written consent and the study was approved by The Regional Committee on Biomedical Research Ethics.

### Study design

This study was a 12 week open randomized parallel diet intervention trial. Sequentially numbered, sealed envelopes containing a computer-generated allocation were used. The intervention consisted of standard MNT and an advice to either: 1) Eat at least two pieces of fruit each day (high-fruit) or 2) eat no more than two pieces of fruit each day (low-fruit). Both groups had two consultations with an experienced registered dietitian at the Outpatient Clinic. One consultation in the beginning and one at the end of the study period. Shortly before consultations in the Outpatient Clinic the subjects had a blood sample drawn at their GP. During the intervention subjects were treated at the GP´s discretion. The primary outcome was change in HbA_1c_. Secondary outcomes were changes in fruit intake, body weight and waist circumference.

### Nutritional intervention

The MNT was given on an individual basis and focused on the individual needs and personal preferences [3]. All overweight subjects were advised to restrict the energy intake. The only difference in the MNT between the groups was the advice concerning fruit intake. The patients were recommended to eat fresh and whole fruit only and to exclude fruit juice, canned and dried fruit from their diet or keep it as low as possible. One piece of fruit was standardized to the amount of a fruit that contained approximately 10 grams of carbohydrate e.g. 100 grams apple, 50 grams banana or 125 grams orange. The patients were given written information and pictures about the amount of the most common fruit that corresponded to one piece.

### Anthropometry measurements

Height was measured at the first visit only. Body weight and waist circumference were measured at both visits. Subjects were weighed barefooted and in light clothing on a calibrated scale. Height was measured using a wall measuring stick scale. Waist circumference was measured horizontally at the level of the umbilicus in a relaxed standing position.

### Biochemical analysis

Blood samples were taken at the subjects GP using standard procedures. The blood samples were analyzed at Department of Clinical Biochemistry, Regional Hospital West Jutland using standard laboratory procedures. HbA_1c_ was analyzed using HPLC.

### Fruit intake

The subjects filled in a weighed 3-day fruit record before and after the intervention. At each visit the fruit intake was estimated using dietary recalls. Portion sizes were estimated and translated to grams using pictures and table values of mean weight for a given standard portion [17,18]. This was compared with the fruit record to avoid errors. Fruit intake was calculated at each visit as mean intake using the 3-day fruit record.

### Physical activity

A self-reported questionnaire was used at each visit to estimate physical activity level [19].

### Statistical analysis

Statistical analysis was performed using Stata statistical software package 11.1 (Stata, College Station, TX, USA). All analyses were performed on an intention-to-treat basis, with a two-sided 0.05 significance level (α=0.05). We used paired t-test to analyze if a variable changed significantly from before to after the intervention. For each outcome we compared mean difference (after - before) between the two groups using an unpaired t-test. For the primary outcome we also used multiple regression analysis controlling for potential confounders. Results are given as mean ± standard error unless otherwise stated.

A sample of 38 subjects in each arm was estimated to detect a difference of 0.7% in HbA_1c_. The sample size was based on achieving an 80% power with α = 0.05, SD = 1.0 and dropout = 10%. The inclusion rate was slower than anticipated. The study was terminated prematurely due to limited time and research funding. Fortunately, the variation in HbA_1c_ was smaller than estimated in the power calculation and there were no dropouts. Therefore the actual power in the study was around 90% to detect a difference of 0.7% in HbA_1c_ between the groups.

## Results

### Baseline characteristics

Recruitment took place from November 2009 to March 2011 with the last visit in June 2011. In total 63 T2DM subjects were included. Baseline characteristics are shown in Table 1. At baseline significantly more subjects were taking oral antidiabetic drugs (OADs) on the high-fruit diet than on the low-fruit diet (22 vs 12; p=0.02). There were no significant differences between the groups for any of the other baseline variables.

**Table 1:** Baseline characteristics of study participants*

	**High-fruit**	**Low-fruit**
	**(n = 32)**	**(n = 31)**
Age (years)	59 ± 12	57 ± 12
Sex (female)	14 (44)	18 (58)
Height (cm)	170 ± 8	169 ± 10
Body weight (kg)	92 ± 17	91 ± 17
BMI (kg/m^2^)	32 ± 5	32 ± 6
Waist circumference (cm)	104 ± 11	107 ± 9
HbA_1c _(%)	6.7 ± 1.2	6.5 ± 1.1
Physical activity level (PAL)	1.7 ± 0,1	1.6 ± 0,1
Fruit intake (g)	194 ± 87	186 ± 82
Duration of diabetes (days)	22 (12–107)	33 (14–54)
Start on OAD prior to intervention†		
1-30 days	10 (45)	7 (58)
31-60 days	6 (27)	3 (25)
> 60 days	6 (27)	2 (17)

*Baseline values are presented as mean ± SD, median (25^th^-75^th^ centile), and number (%). † OAD = oral antidiabetic drugs.

### Compliance to intervention

Based on the fruit records and recalls, the reported fruit intake was altered as expected (Table 2). One subject on the high-fruit diet kept the fruit intake steady just below two pieces a day. The outcomes were unaffected by exclusion of this non-compliant subject.

**Table 2:** Body weight, waist circumference and fruit intake before and after intervention

	**High-fruit**		**Low-fruit**		**Differences between groups**	
	**Before**	**After**	**Before**	**After**	**Means (CI 95%)**	**p-value**
Body weight (kg) †	92.4 ± 2.9	89.9 ± 3.0*	91.2 ± 3.0	89.6 ± 2.9*	−0.9 (−2.2 to 0.4)	0.18
Waist circumference (cm)	103 ± 2	99 ± 2*	107 ± 2	103 ± 2*	−1.2 (−3.0 to 0.5)	0.17
Fruit intake (grams)	194 ± 15	319 ± 24*	186 ± 15	135 ± 7*	175 (119 to 232)	< 0.0001

* Significant difference between before and after.

† Body weight: high-fruit (n = 32), low-fruit (n = 31), waist circumference: high-fruit (n = 27), low-fruit (n = 22), fruit intake: high-fruit (n = 32), low-fruit (n = 31).

### HbA1_c_

**Figure 1:**
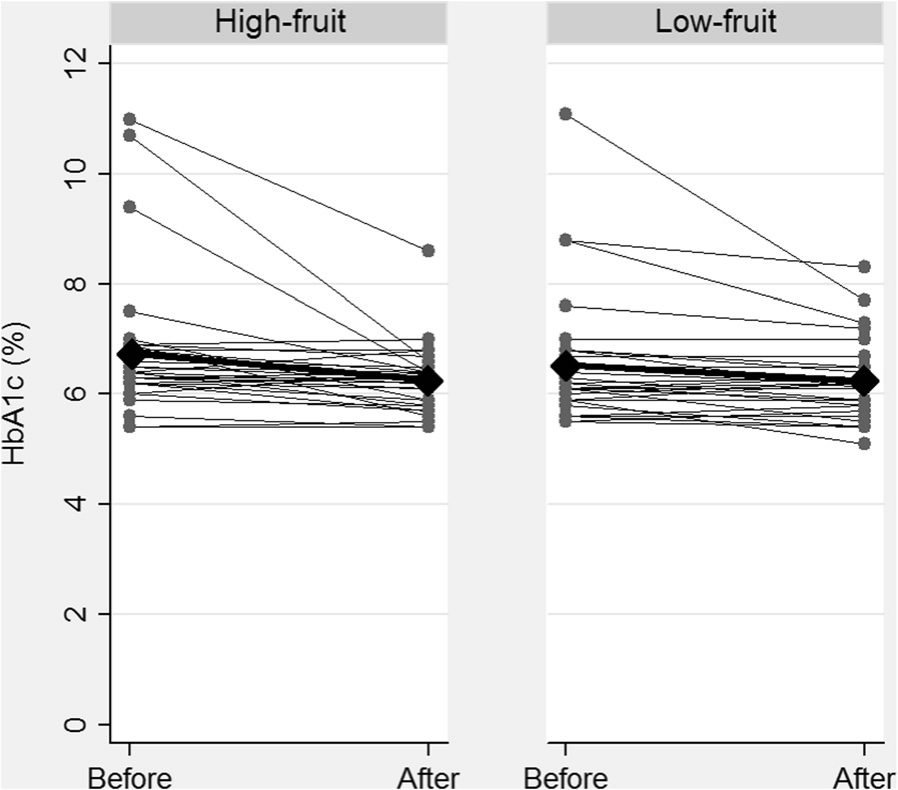
**HbA**_**1c **_**values before and after intervention by groups. **Each thin line represents one subject. Thick line represents mean change.

As expected, there was a significant reduction in HbA_1c_ in both groups. The high-fruit group had a change from 6.74 ± 0.2 to 6.26 ± 0.1% and the low-fruit group a change from 6.53 ± 0.2 to 6.24 ± 0.1%. The reductions were 0.49 ± 0.2 and 0.29 ± 0.1% in the high-fruit and low-fruit diet respectively. There was no significant difference between the groups (Table 3). Adjusting for use of OAD at baseline did not significantly change the result (Table 3). Five subjects (high-fruit = 2; low-fruit = 3) increased OAD dosage during the study period. They had a significantly higher HbA_1c_ at baseline and reduced their HbA_1c_ significantly more during the study (Figure 1).

**Table 3:** **Mean difference between the groups in HbA**_**1c **_**(%)**

	Difference*	**CI 95%**	**p-value**
Unadjusted	0.19	−0.23 to 0.62	0.37
Adjusted for baseline OAD†	0.06	−0.38 to 0.49	0.80

* Difference between the diets (high-fruit (n = 32) – low-fruit (n = 31)) analyzed by multiple regression. † OAD = oral antidiabetic drugs.

### Weight and waist circumference

Both groups had a significant reduction in body weight and waist circumference with no differences between groups (Table 2). The reductions in body weight were 2.5 ± 0.5 and 1.7 ± 0.5 kg in the high-fruit and low-fruit diet respectively. In waist circumference the reductions were 4.3 ± 0.6 and 3.0 ± 0.6 cm in the high-fruit and low-fruit group, respectively. Neither change in body weight (r=−0.07, p=0.61; r=0.17, p=0.19) nor in waist circumference (r=−0.15, p=0.31; r=0.13, p=0.36) was associated with change in fruit intake or change in HbA_1c_ respectively.

### Physical activity

A significant change in physical activity was seen in the high-fruit group (0.07 ± 0.02 PAL; p=0.005) but not in the low-fruit group (0.04 ± 0.03 PAL; p=0.19). However, there was no difference between the groups (0.04 PAL; p=0.31).

### Side effects

One subject on the high-fruit diet, who increased fruit intake from 145 to 310 grams, reported mild gastrointestinal side effects, but the subject remained compliant throughout the study.

## Discussion

Our pragmatic trial demonstrated that, in adults with newly diagnosed T2DM, MNT with an advice to restrict fruit intake resulted in a decreased fruit intake while MNT with an advice to eat more fruit resulted in an increased fruit intake. However this difference in fruit intake did not significantly affect glycemic control, body weight or waist circumference.

To our knowledge, this is the first randomized intervention study examining the effects of dietary advice to restrict fruit intake on glycemic control in T2DM. Most intervention studies with fruit have investigated fruit as a part of a whole diet, fruit mixed with vegetables or only one type of fruit and often as a single meal study e.g. glycemic index studies. Very few intervention studies have tested a variety of fruit over several weeks and none have investigated long-term glycemic control in T2DM subjects.

In our study we found that restriction of fruit intake does not significantly affect HbA_1c_. In a study by Rodriguez et al. 15 obese women randomized to either a low-fruit or a high-fruit diet for 8 weeks a difference between the groups of 550 kJ from fructose was obtained [20]. This is around twice the difference obtained in the present study. The study reported no differences in HOMA, glucose or insulin levels between the groups. In another study by Madero et al. 131 obese subjects were randomized to a low-fructose diet or a moderate-natural- fructose diet for 6 weeks [21]. The groups’ intake of fruit corresponded to approximately 250 and 2200 kJ, respectively in low-fructose and moderate-natural-fructose which corresponds to a difference between the groups about three times as large as in the present study. Significant reductions in HOMA and glucose values were seen within the moderate-natural-fructose group, but no difference between the groups was seen. Cross-sectional studies have shown that fruit intake is not associated [12-15] or inversely associated [16] with HbA_1c_ or other parameters reflecting glycemic control. Further, cohort studies addressing the impact of fruit intake on the incidence of T2DM have shown either no association or an inverse association [22,23]. The evidence, including our present study, therefore suggests that a high fruit intake does not have a negative impact on glycemic control.

We found a tendency towards reduced body weight and waist circumference in the group that ingested most fruit 0.9 (CI 95%; −0.4 to 2.2) kg and 1.2 (CI 95%; −0.5 to 3.0) cm respectively. This corroborates with a few intervention studies. The study by Rodriguez et al. in which the high-fruit group had a significant reduction in waist circumference compared to low-fruit group (5.5 vs. 2.4 cm; p=0.048) [20]. Weight loss was similar in the two groups (6.1 vs. 6.4 kg; p=0.78). In another intervention study 49 obese women were randomized to add either three apples, three pears or three oat cookies to their usual diet for 10 weeks [24]. The total energy and fiber content of the supplements were matched. The two groups with fruit supplements lost significantly more body weight than the group with oat cookies (−0.9 vs −0.8 vs 0.2 kg). In a third study by Madero et al. the moderate-natural-fructose group reduced body weight more than the low-fructose group (4.1 vs 2.9 kg; p=0.02) [21]. A recent review study concluded that in most studies a higher fruit intake has a beneficial effect on body weight and that no studies have found a negative effect [25].

In spite of a difference in fruit intake of about two pieces daily between the groups we did not find any effect on HbA_1c_, body weight or waist circumference. The most likely explanation is that fruit is eaten as a part of a daily diet and therefore when changing the fruit intake it will lead to other changes in the diet. We did not measure total energy intake, but weight and physical activity were similar between the groups and therefore energy intake must have been more or less the same in both groups too. When changing the fruit intake other changes in the diet most likely occur and this would explain that there was no difference in HbA_1c_, body weight and waist circumference despite the significant difference in fruit intake.

Our study has several strengths. It is the first randomized controlled study investigating the relevant scientific question: does fruit intake matter in relation to glycemic control in T2DM subjects? We chose to do this in a “real life” setting. Thus, almost all subjects were fully compliant and there were no drop-outs.

However, our study also has some weaknesses. First, it can be argued that a greater difference in fruit intake between the high and low fruit groups would have resulted in a significant effect, positive or negative. However, we consider a difference of about two pieces of fruit as clinically relevant and we think it reflects a “real life” situation. We admit that testing whether an even higher fruit intake may impact significantly the glycemic control would be interesting, but this was not the intention in this pragmatic trial. Secondly, we did not control (and had no pre-trial intention to do so) the intake of medication. A difference in baseline use of OADs could bias the results. However, adjustments did not significantly change the results (Table 3). Therefore we do not believe it has biased the results. Thirdly, fruit intake and physical activity were self-reported and therefore could have been subject to under or over-reporting. Measurement of biomarkers of fruit intake, e.g. plasma vitamin C and plasma carotenoids would have strengthened the study.

## Conclusions

We conclude that an advice to restrict fruit intake as part of standard MNT in overweight adults with newly diagnosed TDM2 does not improve glycemic control, body weight or waist circumference. Considering the many possible beneficial effects of fruit, we recommend that fruit intake should not be restricted in T2DM subjects.

## Competing interests

The authors declare that they have no competing interests.

## Authors' contributions

All authors were involved in the development of the study protocol. ASC performed subject recruitment, data collection and was responsible for the day-to-day running of the study. ASC and SG performed statistical analysis and drafted the final version of the manuscript. All authors approved and read the final manuscript.

## References

[B1] Danaei G Finucane MM Lu Y Singh GM Cowan MJ Paciorek CJ Lin JK Farzadfar F Khang YH Stevens GA Rao M Ali MK Riley LM Robinson CA Ezzati M National, regional, and global trends in fasting plasma glucose and diabetes prevalence since 1980: systematic analysis of health examination surveys and epidemiological studies with 370 country-years and 2.7 million participants Lancet 2011 378 31 40 10.1016/S0140-6736(11)60679-X 21705069

[B2] Morris SF Wylie-Rosett J Medical Nutrition Therapy: A Key to Diabetes Management and Prevention Clinical diabetes 2010 28 12 18 10.2337/diaclin.28.1.12

[B3] Franz MJ Powers MA Leontos C Holzmeister LA Kulkarni K Monk A Wedel N Gradwell E The evidence for medical nutrition therapy for type 1 and type 2 diabetes in adults J Am Diet Assoc 2010 110 1852 1889 10.1016/j.jada.2010.09.014 21111095

[B4] Bantle JP Wylie-Rosett J Albright AL Apovian CM Clark NG Franz MJ Hoogwerf BJ Lichtenstein AH Mayer-Davis E Mooradian AD Wheeler ML Nutrition recommendations and interventions for diabetes: a position statement of the American Diabetes Association Diabetes Care 2008 31 Suppl 1 S61 78 18165339 10.2337/dc08-S061

[B5] Mann JI De Leeuw I Hermansen K Karamanos B Karlstrom B Katsilambros N Riccardi G Rivellese AA Rizkalla S Slama G Toeller M Uusitupa M Vessby B Evidence-based nutritional approaches to the treatment and prevention of diabetes mellitus Nutr Metab Cardiovasc Dis 2004 14 373 394 10.1016/S0939-4753(04)80028-0 15853122

[B6] Gonzalez-Gallego J Garcia-Mediavilla MV Sanchez-Campos S Tunon MJ Fruit polyphenols, immunity and inflammation Br J Nutr 2010 104 Suppl 3 S15 27 20955647 10.1017/S0007114510003910

[B7] Chong MF Macdonald R Lovegrove JA Fruit polyphenols and CVD risk: a review of human intervention studies Br J Nutr 2010 104 Suppl 3 S28 39 20955648 10.1017/S0007114510003922

[B8] Feeney MJ Fruits and the prevention of lifestyle-related diseases Clin Exp Pharmacol Physiol 2004 31 Suppl 2 S11 13 15649275 10.1111/j.1440-1681.2004.04104.x

[B9] Dauchet L Amouyel P Hercberg S Dallongeville J Fruit and vegetable consumption and risk of coronary heart disease: a meta-analysis of cohort studies J Nutr 2006 136 2588 2593 16988131 10.1093/jn/136.10.2588

[B10] Martinez-Gonzalez MA Lamuela-Raventos RM The unparalleled benefits of fruit Br J Nutr 2009 102 947 948 10.1017/S0007114509353222 19671202

[B11] Aune D Lau R Chan DS Vieira R Greenwood DC Kampman E Norat T Nonlinear reduction in risk for colorectal cancer by fruit and vegetable intake based on meta-analysis of prospective studies Gastroenterology 2011 141 106 118 10.1053/j.gastro.2011.04.013 21600207

[B12] Overby NC Margeirsdottir HD Brunborg C Andersen LF Dahl-Jorgensen K The influence of dietary intake and meal pattern on blood glucose control in children and adolescents using intensive insulin treatment Diabetologia 2007 50 2044 2051 10.1007/s00125-007-0775-0 17687538

[B13] Gulliford MC Ukoumunne OC Determinants of glycated haemoglobin in the general population: associations with diet, alcohol and cigarette smoking Eur J Clin Nutr 2001 55 615 623 10.1038/sj.ejcn.1601233 11464236

[B14] Buyken AE Toeller M Heitkamp G Irsigler K Holler C Santeusanio F Stehle P Fuller JH Carbohydrate sources and glycaemic control in Type 1 diabetes mellitus. EURODIAB IDDM Complications Study Group Diabet Med 2000 17 351 359 10872533 10.1046/j.1464-5491.2000.00283.x

[B15] Panagiotakos DB Tzima N Pitsavos C Chrysohoou C Papakonstantinou E Zampelas A Stefanadis C The relationship between dietary habits, blood glucose and insulin levels among people without cardiovascular disease and type 2 diabetes; the ATTICA study Rev Diabet Stud 2005 2 208 215 10.1900/RDS.2005.2.208 17491696 PMC1783563

[B16] Sargeant LA Khaw KT Bingham S Day NE Luben RN Oakes S Welch A Wareham NJ Fruit and vegetable intake and population glycosylated haemoglobin levels: the EPIC-Norfolk Study Eur J Clin Nutr 2001 55 342 348 10.1038/sj.ejcn.1601162 11378807

[B17] Andersen LT Jensen H Haraldsdottir J Typiske vægte for madvarer Scand J Nutr 1996 40 S129 152

[B18] The Danish Diabetes Association Sund mad - når du har diabetes 2001 Odense

[B19] Johansson G Westerterp KR Assessment of the physical activity level with two questions: validation with doubly labeled water Int J Obes (Lond) 2008 32 1031 1033 10.1038/ijo.2008.42 18392036

[B20] Rodriguez MC Parra MD Marques-Lopes I De Morentin BE Gonzalez A Martinez JA Effects of two energy-restricted diets containing different fruit amounts on body weight loss and macronutrient oxidation Plant Foods Hum Nutr 2005 60 219 224 10.1007/s11130-005-8622-2 16395633

[B21] Madero M Arriaga JC Jalal D Rivard C McFann K Perez-Mendez O Vazquez A Ruiz A Lanaspa MA Jimenez CR Johnson RJ Lozada LG The effect of two energy-restricted diets, a low-fructose diet versus a moderate natural fructose diet, on weight loss and metabolic syndrome parameters: a randomized controlled trial Metabolism 2011 60 1551 1559 10.1016/j.metabol.2011.04.001 21621801

[B22] Carter P Gray LJ Troughton J Khunti K Davies MJ Fruit and vegetable intake and incidence of type 2 diabetes mellitus: systematic review and meta-analysis BMJ 2010 341 c4229 10.1136/bmj.c4229 20724400 PMC2924474

[B23] Hamer M Chida Y Intake of fruit, vegetables, and antioxidants and risk of type 2 diabetes: systematic review and meta-analysis J Hypertens 2007 25 2361 2369 10.1097/HJH.0b013e3282efc214 17984654

[B24] de Oliveira MC Sichieri R Venturim Mozzer R A low-energy-dense diet adding fruit reduces weight and energy intake in women Appetite 2008 51 291 295 10.1016/j.appet.2008.03.001 18439712

[B25] Alinia S Hels O Tetens I The potential association between fruit intake and body weight–a review Obes Rev 2009 10 639 647 10.1111/j.1467-789X.2009.00582.x 19413705

